# The anion study: effect of different crystalloid solutions on acid base balance, physiology, and survival in a rodent model of acute isovolaemic haemodilution

**DOI:** 10.1016/j.bja.2018.01.026

**Published:** 2018-03-21

**Authors:** N.J. Ekbal, P. Hennis, A. Dyson, M. Mythen, M.F.M. James, M. Singer

**Affiliations:** 1 University College London, Bloomsbury Institute of Intensive Care Medicine, London, UK; 2 UCL Centre for Anaesthesia, Critical Care and Pain Medicine, University College London, UK; 3 University of Cape Town, Department of Anaesthesia, Cape Town, South Africa

**Keywords:** acid-base imbalance, bicarbonate, isotonic solutions

## Abstract

**Background:**

Commercially available crystalloid solutions used for volume replacement do not exactly match the balance of electrolytes found in plasma. Large volume administration may lead to electrolyte imbalance and potential harm. We hypothesised that haemodilution using solutions containing different anions would result in diverse biochemical effects, particularly on acid-base status, and different outcomes.

**Methods:**

Anaesthetised, fluid-resuscitated, male Wistar rats underwent isovolaemic haemodilution by removal of 10% blood volume every 15 min, followed by replacement with one of three crystalloid solutions based on acetate, lactate, or chloride. Fluids were administered in a protocolised manner to achieve euvolaemia based on echocardiography-derived left ventrical volumetric measures. Removed blood was sampled for plasma ions, acid-base status, haemoglobin, and glucose. This cycle was repeated at 15-min intervals until death. The primary endpoint was change in plasma bicarbonate within each fluid group. Secondary endpoints included time to death and cardiac function.

**Results:**

During haemodilution, chloride-treated rats showed significantly greater decreases in plasma bicarbonate and strong ion difference levels compared with acetate- and lactate-treated rats. Time to death, total volume of fluid administered: chloride group 56 (3) ml, lactate group 62 (3) ml, and acetate group 65 (3) ml; haemodynamic and tissue oxygenation changes were, however, similar between groups.

**Conclusions:**

With progressive haemodilution, resuscitation with a chloride-based solution induced more acidosis compared with lactate- and acetate-based solutions, but outcomes were similar. No short-term impact was seen from hyperchloraemia in this model.

**Editor's key points**

• Infusing large volumes of crystalloid solutions to replace fluid can be harmful.
• The composition of the ‘ideal’ fluid remains elusive.
• Solutions containing acetate, lactate, or chloride were given to rats subjected to removal of blood volume.
• Some differences in acid-base status were seen but outcomes were similar in all groups.
• Composition of resuscitation fluid may be less important than volume.

While many crystalloid solutions are commercially available, none have the tonicity and electrolyte content that exactly matches that found in normal plasma. Two litres of 0.9% saline (n-saline) may produce significant hyperchloraemic metabolic acidosis.^1^, ^2^, ^3^ This may be associated with pathogenic effects such as a decrease in glomerular filtration,^4^, ^5^ disruption of coagulation,^6^, ^7^, ^8^ and impaired immune function.^9^, ^10^ Conversely, both Hartmann's and Ringer's lactate solutions are hypotonic, with a sodium content well below that of plasma, an osmolarity of 276 mOsm litre^−1^ and osmolality of 257 mOsm litre^−1^.^11^ This may be unsuitable in patients with intracranial pathology because of an increased risk of cerebral oedema^12^ and can also contribute to hyponatraemia.^13^

Various anion combinations—including lactate, acetate, bicarbonate, gluconate, and malate—have been developed in an attempt to produce satisfactory balanced salt solutions. Infusion of an appropriate solution should cause minimal adverse physiological impact, particularly in the setting of severe haemorrhage and resuscitation. The optimal choice of resuscitation fluid is still a matter of debate. The American College of Surgeons recommends the use of normal saline or Ringer's lactate for initial management of shock,^14^ whereas acetate administration is gaining in popularity, particularly in Europe.^15^ Though some animal models support the use of balanced solutions over saline^16^, ^17^ in the treatment of severe haemorrhagic shock, others have been equivocal.^18^ A common problem in the design of these studies is that the resuscitation solution volumes were fixed (×3 bled volume). Determination of intravascular volume in the context of haemodynamic instability can be difficult. Under-resuscitation results in hypoperfusion, whereas fluid overloading increases complications. Thus, fluid responsiveness should ideally be based on dynamic indices measured before and after a fluid bolus.^19^

We investigated the effects of three crystalloid solutions on the balance of plasma electrolytes and acid-base status during isovolaemic haemodilution, with resuscitation volumes based on real-time monitoring of ventricular filling. We were unable to find any prior study that specifically addresses this question using a matched osmolar, chloride-rich solution with concurrent measures of circulatory haemodynamics and regional oxygenation. We hypothesised that solutions containing metabolisable anions (acetate and lactate) would result in less change in plasma bicarbonate (our primary endpoint) and better preserved cardiac function and a longer time to death (secondary endpoints).

## Materials and methods

Methods and results are reported according to relevant ARRIVE guidelines^20^ and compliant with EU directive 2010/63/EU.^21^ All experiments were performed according to local University College London ethics committee approval and Home Office (United Kingdom) guidelines under the 1986 Scientific Procedures Act. Male Wistar rats (∼300 g body weight) were used in all experiments. Before instrumentation, animals were housed in cages of six on a 12 h:12 h light-dark cycle at 20–25 °C. Access to food and water was unrestricted.

Animals were anaesthetised with isoflurane (5% in room air) but remained spontaneously breathing throughout. Adequate depth of anaesthesia was ensured by assessing the stability of arterial pressure and heart rate, and lack of pedal withdrawal response to a nociceptive stimulus. Rectal temperature was maintained at 37°C by placing the animals on a heated mat. Cannulation of the left common carotid artery was performed for blood pressure monitoring, blood sampling, and removal, and of the right internal jugular vein to enable fluid administration for haemodilution. A tracheostomy was sited and connected to a T-piece. The bladder was cannulated for drainage of urine. An oxygen sensor (Oxylite™, Oxford Optronix, Didcot, UK), pre-calibrated by the manufacturer, was inserted into the left vastus lateralis muscle for continuous monitoring of tissue PO_2_ (tPO_2_), as previously described.^22^

**Fig 1:**
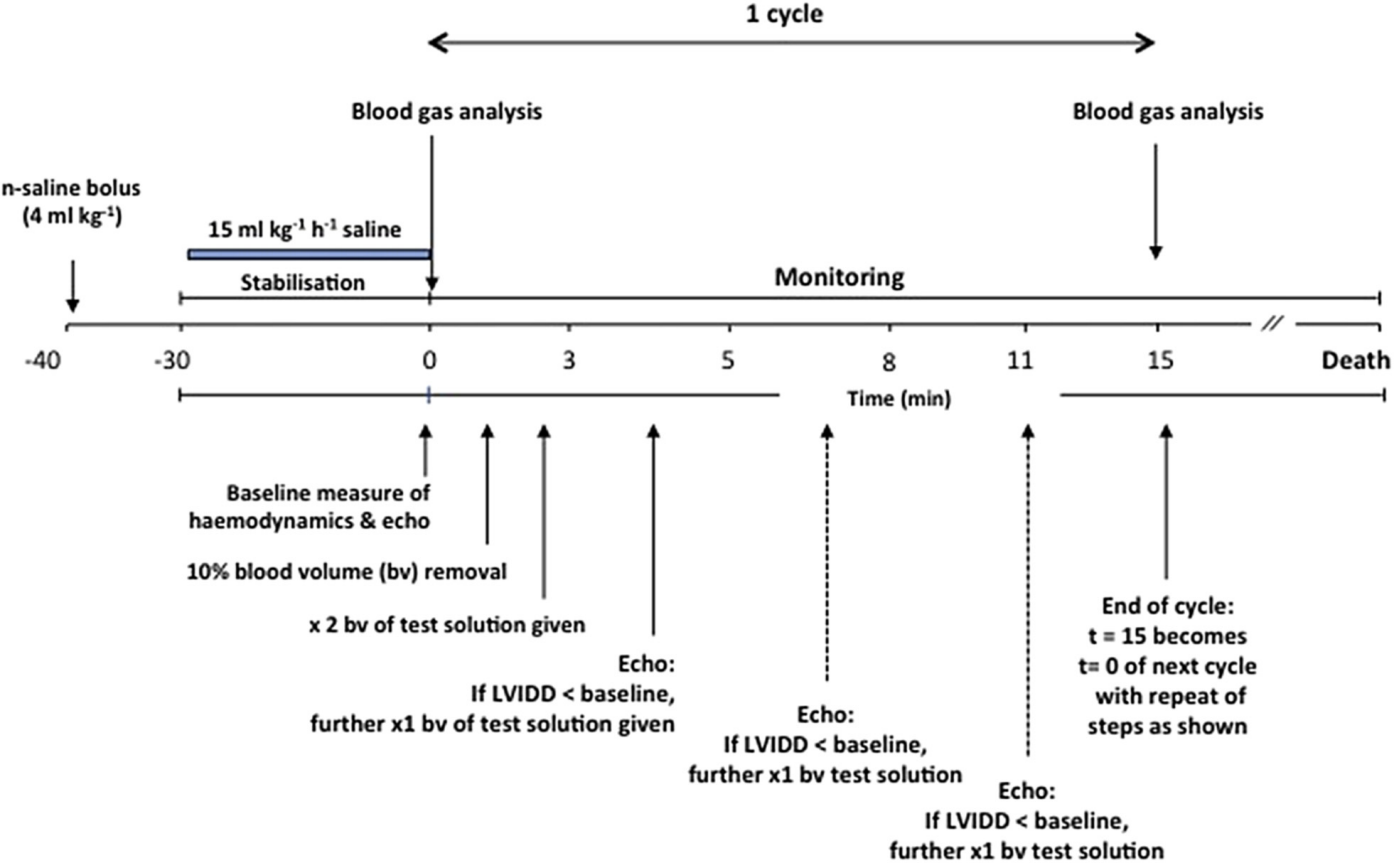
Study plan. Bv, 10% blood volume; LVIDD, left ventricular internal diameter in diastole.

After instrumentation, animals remained anaesthetised with isoflurane throughout the experiment with adequacy of anaesthesia regularly monitored by lack of flexor response to paw pinching. Euvolaemia was achieved by administering 4 ml kg^−1^ n-saline (0.9% sodium chloride; Baxter Healthcare, Thetford, UK) over 10 s followed by a continuous infusion of 15 ml kg^−1^ h^−1^. This regimen had been previously determined from our previous studies where mean arterial blood pressure was not altered by more than 10% and ensured adequate filling at baseline.^23^ The study plan is shown in Fig 1. After a minimum of 30 min stabilisation, the n-saline infusion was terminated, and baseline haemodynamics and tissue oxygenation were recorded (t = 0). Transthoracic echocardiography was performed using a 14 MHz probe scanning at 0–2 cm depth (Vivid 7 Dimension, GE Healthcare, Bedford, UK) by an experienced operator (N.J.E.), in particular, taking note of the left ventricular internal diameter in diastole (LVIDD). Aortic blood flow velocities were determined in the aortic arch using pulsed-wave Doppler. Stroke volume was determined as the product of velocity–time integral and vessel cross-sectional area. Heart rate was determined by measuring the time between cardiac cycles. Ten percent of estimated blood volume was then removed (based on a total of 70 ml kg^−1^) from the arterial line and replaced by twice the volume of a (blinded) test solution. This was repeated at 15-min cycles. The composition of the three test solutions is given in Table 1. Removed blood was sampled at 15-min intervals for plasma ions, acid-base status, haemoglobin and glucose, and derivation of bicarbonate (ABL800FLEX, Radiometer, Copenhagen, Denmark). The animals were randomised to one of three study groups (*n* = 15/group) using an Excel-generated (Microsoft Corp. Redmond, WA, USA) table of random numbers. Sample size was calculated assuming an HCO_3_^−^ difference of −10 mmol litre^−1^ (saline), −7 mmol litre^−1^ (lactate), and −5 mmol litre^−1^ (acetate) with standard deviation (sd) 2.5 mmol litre^−1^; 10 rats per group would be sufficient to demonstrate significant differences in bicarbonate at an alpha = 0.05 and beta = 0.1 (90% power), and 15 per group would be sufficient to show a 20 (10)% difference in fluid volume, and 20 (5)% difference in time to death. A pilot study of five rats suggested that this model was robust and should answer the relevant question.

**Table 1:** Fluid constituents. Ions reported as mEq litre^−1^, osmolarity as mOsm litre^−1^

Fluid	Na^+^	K^+^	Cl^-^	Mg^2+^	Lactate	Acetate	Calculated osmolarity
Chloride solution	140	0	140	1	0	0	282
Lactate solution	140	4	112	1	34	0	291
Acetate solution	137	4	110	1.5	0	34	286.5

Constituents of each study solution (mmol litre^−1^) are shown in Table 1. The chloride (CS) and lactate (LS) solutions were prepared by the Department of Chemistry at UCL and the acetate solution (AS) was supplied by Fresenius−Kabi (Bad Homburg, Germany). The osmolality of the solutions was measured using freezing point depression. Strong ion difference (SID) was calculated as SID = (Na^+^+K^+^)−(Cl^−^+lactate).

To ensure comparable ventricular filling with the different fluids, echocardiography was repeated 3 min after each fluid bolus and filling volume estimated by the LVIDD. If LVIDD was below baseline at 5 min post-fluid bolus, half of the initial fluid dose was given again. This was repeated at 3-min intervals until either LVIDD≥baseline or a maximum of 5× volume of the blood withdrawn was given. This cycle was repeated at 15-min intervals until death. Death was defined as irreversible loss of cardiac contractility on echocardiography. Animals remained anaesthetised throughout.

### Statistics

All haemodynamic and blood gas data are presented as mean and sd unless otherwise stated. Statistical analysis of parametric data was performed using repeated measures two-way analysis of variance with Bonferroni *post hoc* correction. Data for total volume fluid administered are shown as median, inter-quartile range, and range, and were analysed using Kruskal–Wallis testing followed by Dunn's test for *post hoc* comparisons. Survival curves were generated according to the Kaplan–Meier method and were compared with the log-rank test. All statistical analyses were performed using Prism 5.0 software (GraphPad Software, San Diego, CA, USA). Probability values <0.05 were considered significantly different.

## Results

**Fig 2:**
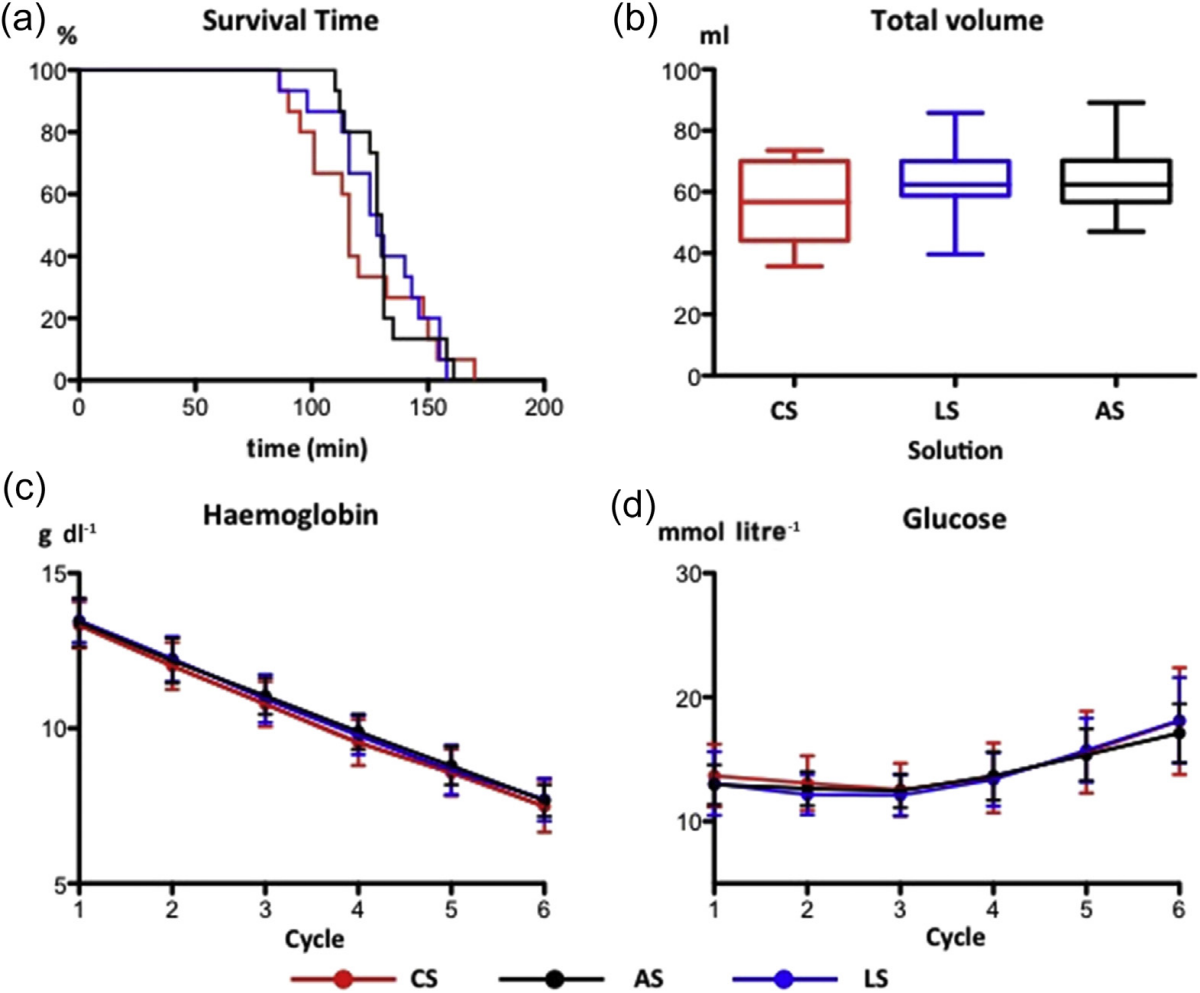
Impact of chloride-, acetate-, and lactate-based fluids on (a) Kaplan–Meier plot of survival time with progressive haemodilution, (b) volume required to maintain ventricular filling (median, inter-quartile and full range), (c) haemoglobin (mean and sd), and (d) serum glucose (mean and sd). AS, acetate-based solution; CS, chloride-based solution; LS, lactate-based solution. *n* = 15 per group.

All groups had similar physiological values after volume optimisation and 30 min stabilisation. Mean survival times between the groups were similar (Fig 2a). No significant difference was seen between the three groups in the total volume of fluid given per animal [CS: 56 (3) ml; LS: 62 (3) ml; AS 65 (3) ml; Fig 2b].

As the first animal died before Cycle 7, the beginning of Cycle 6 was defined as the last possible point for comparison of the haematological, biochemical, cardiovascular, and tissue oxygen parameters between the three groups. For clarity, all subsequent figures are shown up until this time point. There was a stepwise decrease in haemoglobin (Fig 2c) and an increase in glucose (likely stress-related) (Fig 2d) that was consistent between groups.

**Fig 3:**
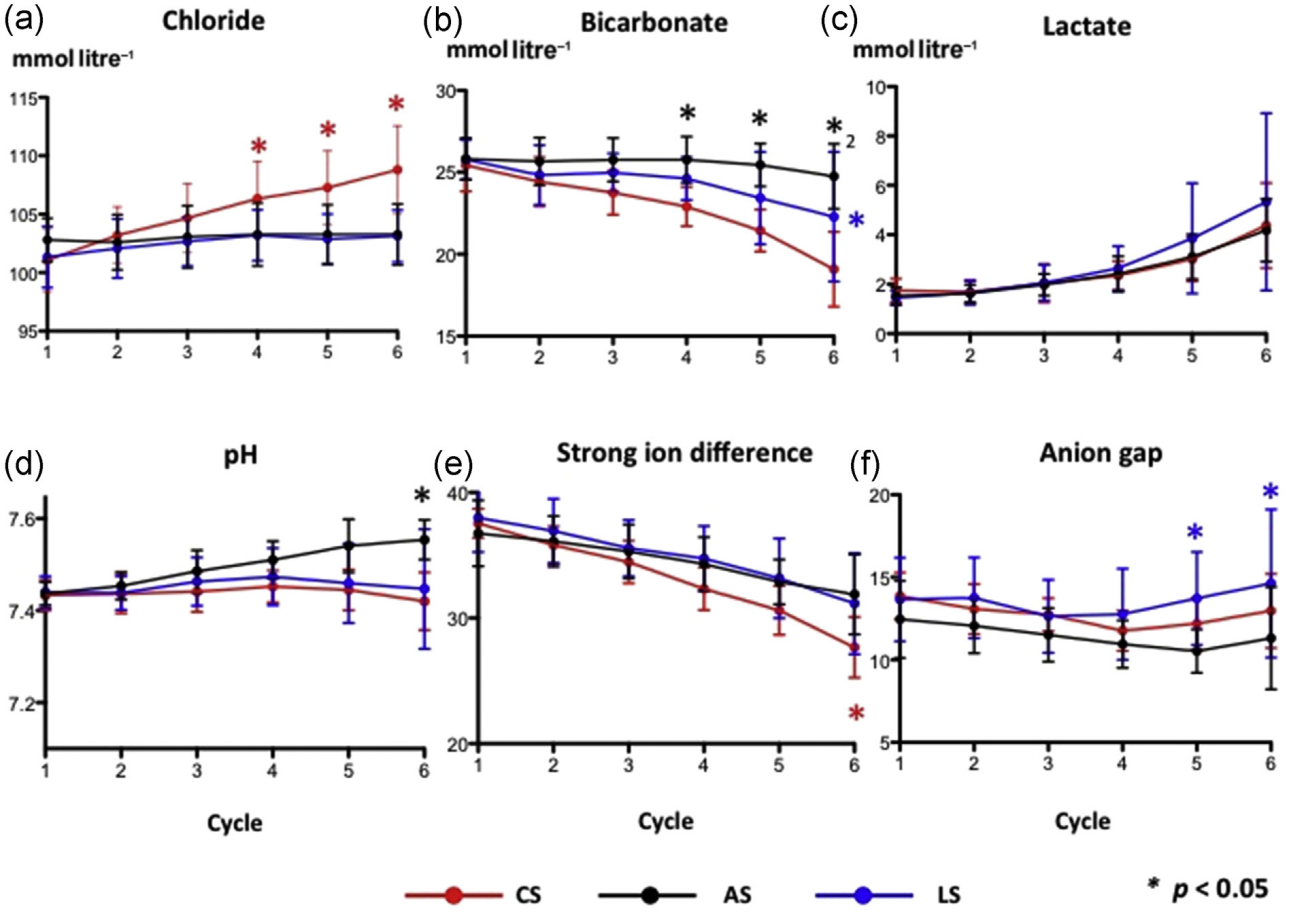
Impact of chloride-, acetate-, and lactate-based fluids on serum levels of (a) chloride, (b) bicarbonate, (c) lactate, (d) arterial pH, (e) strong ion difference (SID), and (f) anion gap. All data are mean and sd. *Significant differences between groups, *P* < 0.05. AS, acetate-based solution; CS, chloride-based solution; LS, lactate-based solution. *n* = 15 per group.

Plasma chloride rose progressively in the CS group but remained unchanged in the LS and AS groups (Fig 3a). There was a corresponding decrease in plasma bicarbonate in the CS group, whereas bicarbonate fell later in the lactate group but was unchanged at the end of Cycle 6 in the acetate group (Fig 3b). Arterial lactate concentrations increased with progressive haemodilution. While the increase was greater in the LS group, this did not differ significantly from the other two groups.

**Fig 4:**
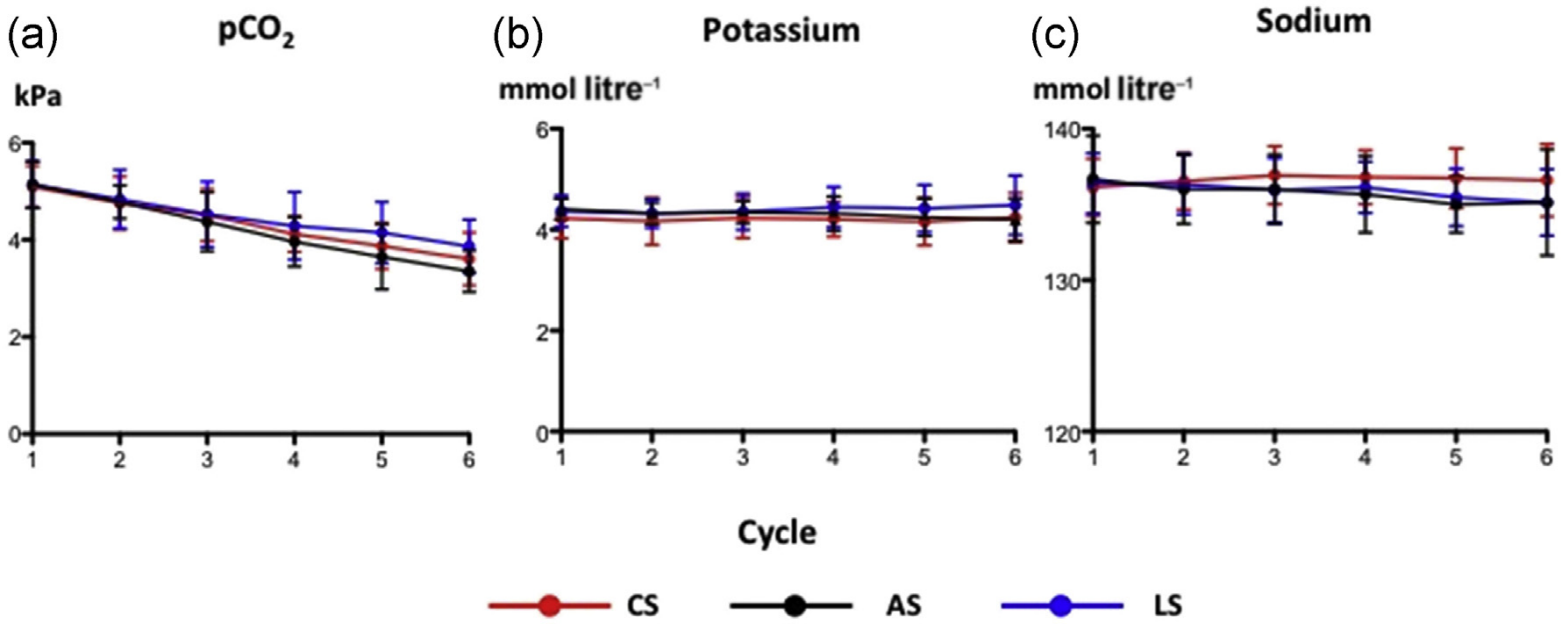
Impact of chloride-, acetate-, and lactate-based fluids on (a) arterial carbon dioxide tension, (b) serum potassium, and (c) serum sodium. All data are mean and sd. AS, acetate-based solution; CS, chloride-based solution; LS, lactate-based solution. *n* = 15 per group.

Despite significant decreases in bicarbonate and SID with chloride (Fig 3d and e), pH remained constant because of compensatory hyperventilation and hypocapnia (Fig 4a). Sodium and potassium concentrations stayed normal throughout (Fig 4b and c). The arterial pH, however, rose markedly with acetate, despite a decrease in SID and no change in anion gap (Fig 3f), but remained unchanged with lactate despite a decrease in SID. Of note, PaCO_2_ remained similar between groups while the calculated anion gap only showed a late increase with lactate that was significantly different with respect to acetate but not bicarbonate.

**Fig 5:**
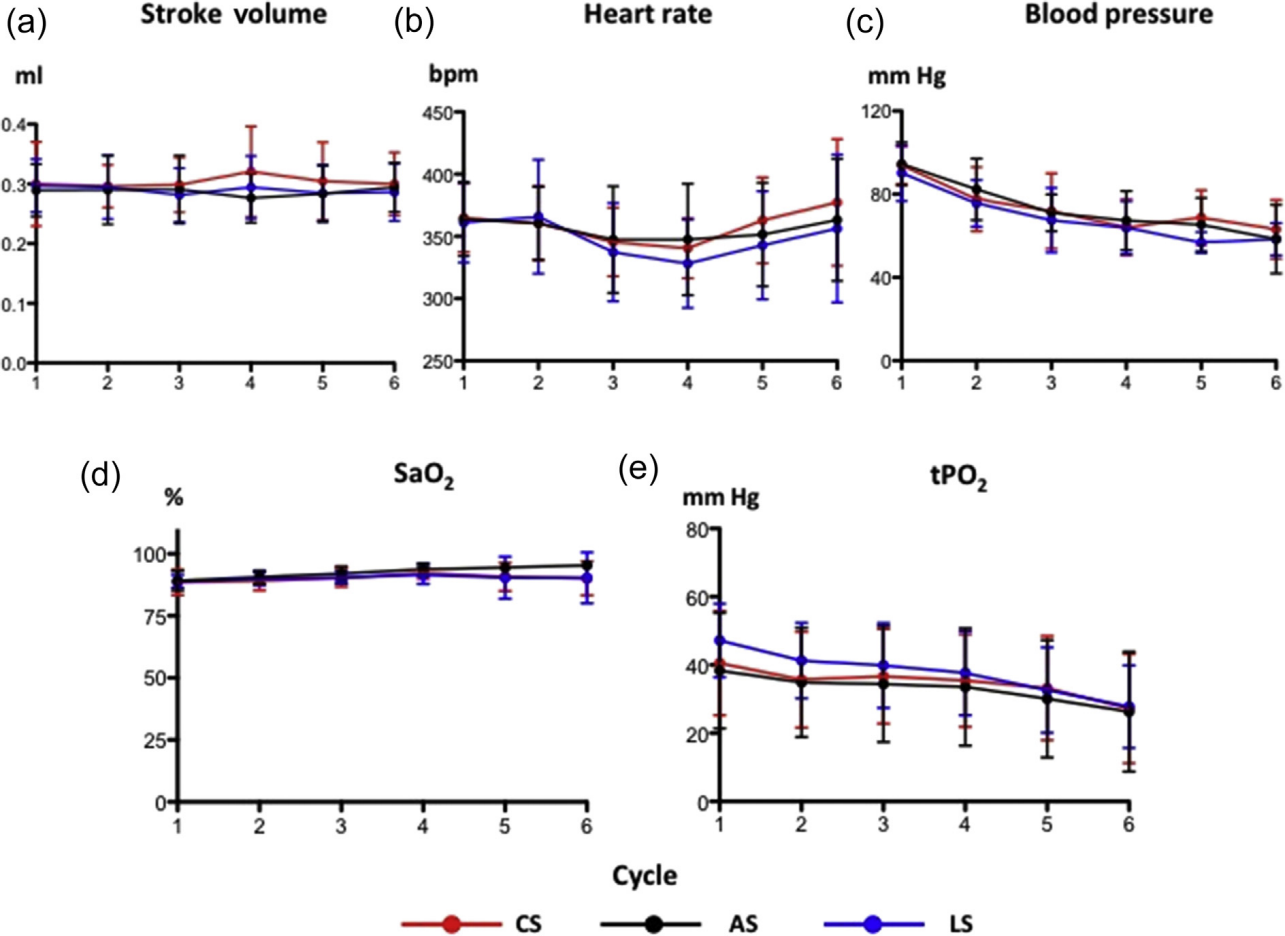
Impact of chloride-, acetate-, and lactate-based fluids on (a) stroke volume, (b) heart rate, (c) blood pressure, (d) arterial oxyhaemoglobin saturation, and (e) skeletal muscle tissue oxygen tension. All data are mean and sd. AS, acetate-based solution; LS, lactate-based solution; S, chloride-based solution; tPO_2_, muscle tissue oxygen tension. *n* = 15 per group.

With increasing haemodilution, there was a stepwise decrease in systemic blood pressure (Fig 5c), however, stroke volume and heart rate remained constant because of the fluid replacement (Fig 5a and b). Skeletal muscle tissue PO_2_ gradually decreased (Fig 5e), consistent with the progressive decrease in oxygen supply to the muscle with the haemodilution. In terms of haemodynamics, there was no between-group difference in any variable measured.

## Discussion

A balanced crystalloid solution should ideally contain constituent electrolytes that impose no physiological stress or disturbance to normal physiology. The challenge in developing a more appropriately balanced crystalloid solution revolves around difficulty in providing a suitable anion to balance the cation content, as a significant amount of the plasma anionic buffer is normally provided by albumin. Using an animal model of progressive haemodilution, we performed a comprehensive assessment of the physiological and biochemical effects of resuscitation with fluids containing the most widely used anions: chloride, lactate, and acetate combined with similar concentrations of sodium. Despite distinct biochemical effects produced by the three separate crystalloids, there were no differences in haemodynamics, tissue oxygenation, total volume of fluid required, or time-to-death.

Significant separation of plasma chloride was observed early in the chloride group, as expected. Although this did not cause a decrease in arterial pH, a significant difference in SID was seen. Acetate maintained bicarbonate concentrations albeit with a significant metabolic alkalosis compared with the other two groups. Acetate is metabolised by several organs, but mainly by muscle where it is converted to the central intermediary metabolite, acetyl coenzyme A. In contrast, lactate is mainly converted to either glucose or bicarbonate in the liver; in conditions of disrupted hepatic perfusion, its metabolism may be disturbed. This may account for both the greater decrease in bicarbonate seen with lactate compared with acetate, and the lack of a pH alkalisation effect seen with infusion of lactate. The reduced bicarbonate concentration explains the raised anion gap seen in the lactate group, while the anion gap is maintained in the chloride group because of the induced hyperchloraemia. There was no inter-group difference in serum glucose, thus the effect of organic anions (acetate and lactate) on glucose metabolism described elsewhere^24^ was not seen.

The differences in acid-base status did not impact on either survival or the total volume of resuscitation fluid infused. In a rodent model of severe haemorrhagic shock, acetated Ringer's solution (27 mM acetate) improved metabolic acidosis and prolonged median survival compared with lactated Ringer's or n-saline.^18^ Tissue injury, assessed by plasma enzyme activities, was most pronounced with lactated Ringer's, medium with n-saline, and least with acetated Ringer's solution. In contrast, in a swine model of severe haemorrhage, resuscitation with Ringer's lactate solution provided the best survival rate followed by n-saline and then Plasmalyte (pH 7.4, 27 mM acetate), yet pH was highest with the AS.^25^ On the other hand, a comparison of Ringer's lactate and n-saline in another pig haemorrhage model showed no difference in survival but only a possible vasodilatatory effects of n-saline.^26^ A recent study compared Ringer's lactate, Ringer's acetate, PlasmaLyte, and n-saline in a rat model of haemorrhagic shock in the presence or absence of a 70% partial liver resection.^27^ Arterial bicarbonate content and pH were higher in animals receiving Ringer's acetate and PlasmaLyte, although haemodynamics (including renal blood flow and blood pressure) and renal oxygenation were similar across all four groups.

Observational data in patients suggested that restricted use of chloride-rich fluids was associated with a decreased risk of renal complications.^28^ However, a recent prospective randomised multi-centre trial in intensive care unit patients failed to show any effect on renal injury or other outcomes comparing a balanced crystalloid solution *vs* n-saline, although the volumes of crystalloid administered were rather small.^29^ Prophylactic addition of sodium bicarbonate during cardiac surgery to alkalinise the urine failed to prevent acute kidney injury, and was associated with an increase in mortality.^30^ Our results support a recent Cochrane analysis of randomised trials comparing balanced with unbalanced fluid solutions for use in surgery, which concluded that saline-based fluids were equivalent to the use of balanced solutions in terms of clinical outcome.^31^

The recognition of a metabolic acidosis after administration of chloride-rich solutions often induces clinical concern, with reflex administration of further fluid in the mistaken interpretation that the acidosis signifies ongoing hypovolaemia rather than from iatrogenic hyperchloraemic aetiology. Whether one is a proponent of the classical Henderson–Hasselbalch equation or the Stewart hypothesis, the use of acid-base balance as a specific surrogate of hypovolaemia is flawed and may lead to adverse effects. All crystalloids lack oxygen-carrying capacity,^32^ and may have potential pro-inflammatory and oxidative effects.^33^ Ideally, as used in this study, minimally-invasive, real-time dynamic haemodynamic monitoring (e.g. using change in stroke volume or end-diastolic ventricular diameter) should assess the impact of manoeuvres that alter venous return. This permits accurate titration of fluid that should minimise problems related to fluid under- or overload.

### Limitations

We used a short-term model of progressive haemodilution to assess the impact of the different crystalloid fluids, with no attempt to return shed blood. In clinical practice, ongoing severe haemorrhage would prompt transfusion of blood. Whether this would result in any difference remains to be seen. However, our objective was to determine the acid-base effects of the three crystalloids in the absence of any confounding variables induced by blood transfusion. The animals were anaesthetised with isoflurane, which carries potential vasodilatatory and negative inotropic consequences that may impact upon tissue perfusion. However, experience over many years with this and similar models has revealed isoflurane to offer the greatest degree of cardiorespiratory stability in comparison with i.v. agents. Urinalysis was not performed as the animals became oliguric and then anuric early in the haemodilution protocol. In view of the short-term nature of the study, urea or creatinine was not measured. While differences likely exist between rat and man in terms of physiology, blood buffering capacity, and blood composition, comparison data in the stress environment are sparse. Similarly, differences in handling of the different anions also needs to be elucidated. Of note, acid-base homeostasis is remarkably similar across all non-hibernating mammalian species. Rats adequately reflect the human response to exercise,^34^ and we consider the response to haemorrhage would likely be similar. Finally, it would be of interest to repeat the study with the animals under heavy sedation with/without paralysis and controlled mechanical ventilation to maintain fixed PaCO_2_ values, acknowledging the impact of positive airways pressures and decreased sympathetic activation on the stress response.

## Conclusions

We compared resuscitative effects of chloride-, acetate- and lactate-containing crystalloid solutions in a rodent model of sequential haemodilution. The CS was associated with hyperchloraemia and the largest decreases in bicarbonate and SID, while the AS maintained serum bicarbonate but induced a significant metabolic alkalosis. However, all solutions had equivalent effects on haemodynamics and tissue oxygenation, volumes required to maintain diastolic filling at baseline levels, and time-to-death. Our results from the current study suggest that the metabolisable anions, acetate, and lactate, did not have an impact on either survival or total volume administered when compared with chloride. Regardless of composition, the main challenge in fluid therapy is the identification of the optimal volume that needs to be infused to correct tissue hypoperfusion adequately, but avoid overload. This should be the main goal when considering safe administration of resuscitation fluid.^35^

## Authors' contributions

Designed the study: A.D., M.F.M.J., M.M., M.S.

Performed the experiments: N.J.E., P.H., A.D.

Interpreted the data: N.J.E., M.F.M.J., M.S.

Wrote the initial manuscript: N.J.E., M.S.

Comment and contribution to the initial manuscript: A.D., P.H., M.F.M.J., M.M.

## Declaration of interest

M.M. is a member of the Editorial Board of the *British Journal of Anaesthesia* and a consultant for Edwards Lifesciences and Deltex. M.M. has organised educational meetings that have received financial support from Fresenius–Kabi (www.ebpom.org). M.S. is developing a bladder tissue oxygen sensor with Oxford Optronix (clinical study funded by Department of Health and Wellcome Trust) and is a consultant for Deltex Medical and New B Innovation. M.F.M.J., M.M., and M.S. have received speaker fees from Fresenius.

## Funding

UK Medical Research Council Studentship Award (to N.J.E.). Fresenius–Kabi provided an unrestricted grant to support the study. Oxford Optronix provided the tissue PO_2_ probes. M.S. is a National Institute of Health Research (NIHR) Senior Investigator. The work was performed at University College London Hospitals/University College London (UCLH/UCL) which receives support from the NIHR Biomedical Research Centre funding scheme.
